# Plant Metabolomics: An Overview of the Role of Primary and Secondary Metabolites against Different Environmental Stress Factors

**DOI:** 10.3390/life13030706

**Published:** 2023-03-06

**Authors:** Uzma Salam, Shakir Ullah, Zhong-Hua Tang, Ahmed A. Elateeq, Yaseen Khan, Jafar Khan, Asif Khan, Sajid Ali

**Affiliations:** 1 Key Laboratory of Plant Ecology, Northeast Forestry Universit y, Harbin 150040, China; 2 Horticulture Department, Faculty of Agriculture, Al-Azhar University, Nasr City, Cairo 11754, Egypt; 3 Key Laboratory of Plant Nutrition and Agri-Environment in Northwest China, Ministry of Agriculture, College of Natural Resources and Environment, Northwest A&F University, Xianyang 712100, China; 4 Laboratory of Phytochemistry, Department of Botany, University of São Paulo, São Paulo 05508-010, Brazil; 5 Department of Horticulture and Life Science, Yeungnam University, Gyeongsan 38541, Republic of Korea

**Keywords:** metabolomics, tolerance, metabolic responses, biotic stress, abiotic stress, metabolites variation

## Abstract

Several environmental stresses, including biotic and abiotic factors, adversely affect the growth and development of crops, thereby lowering their yield. However, abiotic factors, e.g., drought, salinity, cold, heat, ultraviolet radiations (UVr), reactive oxygen species (ROS), trace metals (TM), and soil pH, are extremely destructive and decrease crop yield worldwide. It is expected that more than 50% of crop production losses are due to abiotic stresses. Moreover, these factors are responsible for physiological and biochemical changes in plants. The response of different plant species to such stresses is a complex phenomenon with individual features for several species. In addition, it has been shown that abiotic factors stimulate multi-gene responses by making modifications in the accumulation of the primary and secondary metabolites. Metabolomics is a promising way to interpret biotic and abiotic stress tolerance in plants. The study of metabolic profiling revealed different types of metabolites, e.g., amino acids, carbohydrates, phenols, polyamines, terpenes, etc, which are accumulated in plants. Among all, primary metabolites, such as amino acids, carbohydrates, lipids polyamines, and glycine betaine, are considered the major contributing factors that work as osmolytes and osmoprotectants for plants from various environmental stress factors. In contrast, plant-derived secondary metabolites, e.g., phenolics, terpenoids, and nitrogen-containing compounds (alkaloids), have no direct role in the growth and development of plants. Nevertheless, such metabolites could play a significant role as a defense by protecting plants from biotic factors such as herbivores, insects, and pathogens. In addition, they can enhance the resistance against abiotic factors. Therefore, metabolomics practices are becoming essential and influential in plants by identifying different phytochemicals that are part of the acclimation responses to various stimuli. Hence, an accurate metabolome analysis is important to understand the basics of stress physiology and biochemistry. This review provides insight into the current information related to the impact of biotic and abiotic factors on variations of various sets of metabolite levels and explores how primary and secondary metabolites help plants in response to these stresses.

## 1. Introduction

Comprehensively, biotic and abiotic stresses negatively affect crop production and cause a marked decrease in annual crop yield, i.e., qualitative and quantitative [1,2]. Recently, biologists, especially agriculturists, need to find an alternative way to deal with biotic and abiotic stresses such as herbivores, insects, and pathogens, as well as salinity, trace metals (TM) contamination, drought, and extreme temperatures [3,4] respectively. All these stresses affect the physiological and morphological aspects, such as the hindering of the functional groups of important molecules, e.g., enzymes, polynucleotides, transport systems for substantial ions and nutrients, as well as the growth and metabolic activities of plants [5,6]. However, to cope with these stresses, plants adopt several mechanisms, including metabolomics, transcriptomics, proteomics, and genomics, individually or in combination. The plant metabolome consists of the following two kinds of metabolites: primary and secondary metabolites. Primary metabolites are essential for the proper growth and development of plants and microorganisms. On the contrary, secondary metabolites are formed near the stationary phase of growth and have no direct role in growth, reproduction, and development. The metabolic profiling of primary and secondary metabolites provides extensive knowledge of biochemical processes that occurs in plant metabolism [7]. 

Modern research endorsed the purpose of several important genes, metabolites, proteins, and molecular systems that induced plant reactions to drought, salt stress, cold, TM, heat, and certain other biotic and abiotic factors [8,9]. Metabolomics analyses have become an influential tool to monitor plants’ responses to different environmentally stressed conditions [10]. Therefore, the findings of such studies give an understanding of the working of plants in definite circumstances, which are considered an important part of enlightening the molecular processes in responses to various stress conditions [11]. An appropriate data analysis, detection, identification, and evaluation of these metabolites are possible with the help of advanced metabolic tools such as gas chromatography-mass spectrometry (GC-MS), liquid chromatography-mass spectrometry (LC-MS) nuclear magnetic resonance (NMR) [12]. 

Furthermore, it is estimated that biotic and abiotic stresses are responsible for more than 50% of crop losses in the world [13,14]. The findings of Bayer in 2008 demonstrated that crop losses caused by abiotic stressors were significantly higher than by biotic factors [15]. However, the exact loss of crop yield depends on the plant’s developmental stage and the intensity and duration by which various stresses occur [16]. Among other stresses, salinity affects more than 800 million hectares of land—nearly 50% of the total irrigated area, which provides about 33% of the world’s food [17,18]. In the same way, drought also causes a loss of more than 50% of the average yield of crops [19]. Subsequently, other studies indicated that abiotic factors, such as temperature (low or high), salinity, and drought, significantly decreased plant production if existing alone or in combination [20]. Interestingly, another concern is the aggregation of reactive oxygen species (ROS), which is produced by excessively stressed accumulators of cadmium (Cd), chromium (Cr), lead (Pb), zinc (Zn), and copper (Cu) that can cause oxidation and dysfunction of biological molecules, hence disturbing certain physical and biological processes in plants [3,21]. Optimizing metabolic flux by the organellar electron transport chain (ETC) is essential in reducing oxidative stress [3]. Consequently, keeping the redox state of a cell is another essential issue that provides the decreasing power necessary for the foraging of ROS [22]. 

Therefore, there is a need for novel, easy, inexpensive, ecologically friendly, and robust crop types that can be conceived by cross-breeding or genetic engineering [23]. For example, recently, different wheat, rice, barley, maize, and other economically crucial varieties of crop plants have been considered very necessary than model plants [24,25]. However, the development of some modern ‘omics tools, such as genomics, proteomics, transcriptomics, and metabolomics, has rationalized the research of crop plants and abetted the complete study of acquaintances concerning biological components and plant breeding [26]. In this concern, metabolomics gives the possibility to accelerate the selection of superior breeding stock and the screening of elite crop types [27]. Primary and secondary metabolites, with their functional diversity, play an important role in fine-tuning the environmental stress tolerance and productivity in crops. Understanding plant behavior under multiple environmental stressors is one of the ways to deal with agricultural sustainability [20]. In this piece of work, more than 200 published works were considered to provide an overview of the role of primary and secondary metabolites against several abiotic and biotic stressors.

## 2. Instrumentation Applied in Metabolomics Studies

The identification of different classes of metabolites in plants is largely based on using hyphenated mass spectrometric methods to chromatographic equipment and electrophoretic approaches [28]. Choosing an appropriate ionization technique and analyzer type for metabolite analysis is important in a mass spectrometer [29]. Through the study of mass spectrometry (MS), ionized molecules are calculated. Similarly, mass-to-charge ratio values (*m*/*z*, m-mass, or z-charge) of the produced ions are assessed with the precision of one mass unit and to the fourth decimal point, small or high-resolution mass spectra, after elimination in the MS analyzer. The use of a high-resolution mass analyzer permits the accomplishment of the elemental composition of the identified ions existing in mass spectra. At first, it is probable to estimate the elemental composition and molecular mass of the molecules from enumerated *m*/*z* values for protonated [M + H]^+^ and deprotonated molecules [M − H]^−^. The clear documentation of compounds is highly dependent on the applied MS system. MS machines designed with electrospray ionization (ESI) and matrix-assisted laser desorption or ionization (MALDI) source could be utilized. The ionization of MALDI can be joined to one or two unified times of flight analyzer (TOF and TOF/TOF). The source of ESI works well through quadrupole (Q), ion trap (IT), time of flight (TOF) analyzer, and a mixture of them. The maximum resolution in the mass analyzer could be attained by ion cyclotron resonance through Fourier Transformation Instruments (FT ICR MS) when the ESI is employed as an ionization system.

Moreover, designing the experiment according to the Metabolomics Standard Initiative (MSI) is also crucial, which endorses defined measures for the right biological materials preparation, procedures of metabolite extraction, and analytical protocols [30]. Following the regulations that have been stipulated, a sufficient number of sample replications and the conditions under which plant development should occur to be investigated and defined [31]. Similarly, the control of MS parameters in mass spectra registration is necessary. Such data deliver environments for suitable documentation and quantification of metabolites and consistent statistical quantification [32]. 

After employing this method, different statistical calculations could be performed to determine the metabolites’ capacities that allow the defining changes of a specific compound in definite situations [33]. The number of primary and secondary metabolites in a single organism may range from several hundred to tens of thousands, with little variation across orders of magnitude in concentration. Some strategies developed for metabolites analysis include metabolic profiling, metabolic fingerprinting, and target analysis [34]. 

Metabolic profiling is expected a simultaneous measure of a set of metabolites in a sample. Several analytical techniques can be used for metabolic profiling, such as (GC-MS), (LC-MS), and (NMR). To date, GC-MS is the most advanced analytical approach to metabolic profiling in plants [35]. Using GC-MS, it is possible to recognize several hundred compounds belonging to various classes, including sugar, organic acids, amino acids, alcohols, amines, and fatty acids. Similarly, LC-MS provides a better alternative for non-volatile compounds. The importance of LC-MS is increasing in metabolomics, especially after the adoption of ultra-performance liquid chromatography technology that can increase separation efficiency and decrease analysis time [36]. Substantially, NMR spectroscopy offers an entirely different analytical technique compared to MS-based approaches. The sensitivity of the NMR technique is much lower than MS-based techniques; however, the structural content information, reproducibility, and computable aspect could be superior to them [37]. Moreover, the preparation of the sample is simple, more convenient, and non-destructive measurement may possible. These properties of NMR make it an ideal tool for the identification of metabolites through metabolic profiling [38].

## 3. Workflow of Plant Metabolomics Analysis

The metabolomics of plants is very complex and varied in their chemical structure. Extensive identifications and a wide range of metabolic depictions could be attained with the arrangement of two or more metabolomics approaches and analytical methods, with the difference in extraction protocols [39]. Metabolomics analyses comprise the following three key tentative methods: (1) sample preparation, (2) data gaining, and (3) the identification of compounds by using the statistical analysis of the data. The preparation of the sample is a key step because it can contribute to the identification of a wide range of metabolites, which is comprised of tissue collecting, drying, or quenching, and metabolite extraction for analysis (derivatization) [40]. Thus, care should be taken in this step to avoid engaging in undesirable variation that can significantly disturb the analysis results. Many methods of enzyme quenching, such as drying, enzyme inhibitors and acids, and high meditations of organic solvents, could also distress the analysis and identification [41]. 

Plant metabolites are structurally different with high complications, such as dissimilar size, solubility, explosive nature, separation, amount, and stability [42,43]. The extraction method of metabolites relies on varied factors such as the type of plant organs, physical and chemical properties of the targeted metabolites, chemical structure, and the solvent used [44]. Generally, metabolite extraction methods include solvent extraction, supercritical fluid extraction, solid-phase extraction, and sonication [45]. Moreover, other methods are used to extract the essential oils, such as hydrodistillation, vapor-hydrodistillation, vapor-distillation, hydro diffusion, organic solvent extraction, and cold pressing [46]. Though, it is critically essential to evaluate metabolite extraction methodologies for a precise metabolite extraction study because a solvent composition that is good for one chemical class may not be suitable for another chemical class. Moreover, this could not be appropriate for extracting large numbers of metabolites from a specific tissue. So, it is important to understand and monitor the effects of the applied solvent treatment on the sample's metabolic content and profile obtained [47]. 

The measurement of complex metabolites needs an advanced analytical platform for sample analysis. Every platform’s range has a particular constraint, maybe in selectivity or sensitivity [48]. The selection of the analytical platform relies on the study initiated, the group of compounds, and their physiochemical properties, such as polarity, solubility, volatility, and concentration levels [49]. Additionally, one issue is that metabolites occur in a wide dynamic range of concentrations such as nanomolar and millimolar in the plant body. Subsequently, another problem is that not every metabolite is present in each tissue [50].

**Figure 1:**
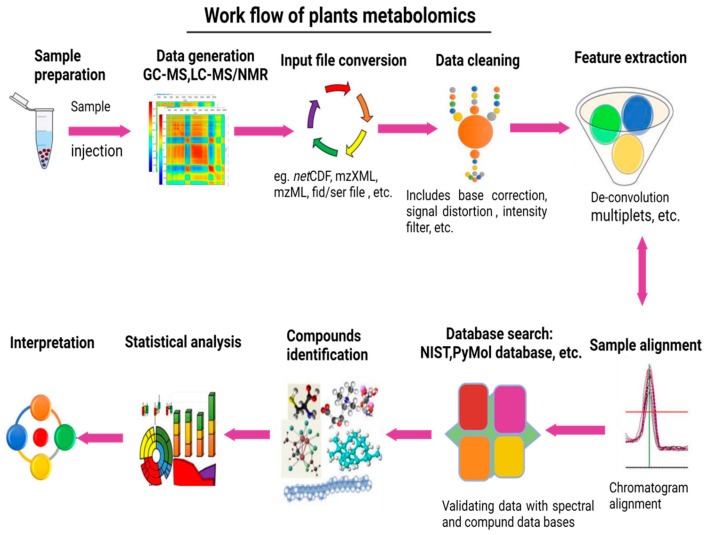
Respective illustration of the processes involved in plant metabolomics analysis of GC–MS, LC-MS, CE-MS, and NMR-based chromatography.

However, the most applied metabolomics approaches in analytical studies are liquid or gas chromatography synchronized with mass spectrometry (LC/GC-MS) and nuclear magnetic resonance spectrometry (NMR) [51]. Subsequently, another report [52] demonstrated an integrated technique that combines metabolites extraction and analysis with proteomic and RNA from a single sample that permits the immediate inquiry of all molecular levels and examines their interrelation and co-variance structure [53]. Consequently, biochemical regulation could result in the co-variance design of molecular dynamics in a cellular system [54]. In the context of metabolomics, the block diagram (Figure 1) of a typical experiment shows the following key steps:

1. Sample collection and organization;
2. Metabolites extraction;
3. Derivatization and separation;
4. Data acquisition;
5. Data analysis;
6. Metabolites identification;
7. Data submission to public repositories.

## 4. Metabolomics for Plant Stress Responses

Metabolomics is the scientific study of the set of metabolites present within an organism, plant cell, or tissue [55]. However, plant stress is any amendment in the growth and developmental conditions that distracts metabolic homeostasis and needs to modify the metabolic pathways in a process generally designated as acclimatization [35]. Over the last decade, metabolomics has developed promptly and is recognized as the prevailing technology in changing climatic conditions and assessing or elucidating testing phenotypes in assorted living systems [56]. Substantially, it may contribute to studying stress biology in plants or other organisms by recognizing various molecules, such as by-products of stress metabolism and compounds of stress signal transduction and related to the plant acclimation responses [52]. Their application has been driven in several fields, including medicinal, imitation biology, or analytical molding of plants, animals, and microscopic organisms [57]. 

**Figure 2:**
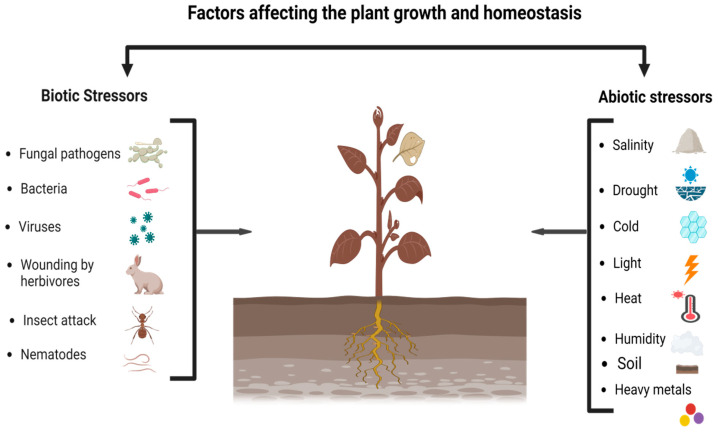
Environmental stresses of biotic and abiotic factors affecting the growth and homeostasis of plants.

Additionally, to the applicability of other fields, nowadays metabolomics could also be used on a large scale in the assortment procedure of plants and resistant to the varying environmental states. Different findings revealed that drought stress, salinity, extreme temperature, and soil flooding could cause significant instabilities in the pattern of plant metabolome [22]. Metabolomics signifies the ultimate omic’s level in a living system or reveals modifications in the traits of an organism or function. Different findings show the study of metabolomics under several environmental abiotic stresses, such as temperature [58], salinity and drought [59], and soil flooding [60]. In the same way, various metals and metalloids including, sulfur [61], phosphorus [62], oxidative stress [63], TM [64], and the combination of other several stress factors [65] in plants (Table 1). Various environmental factors that could negatively disturb the homeostasis and growth of plants are shown below (Figure 2).

### 4.1. The Response of Primary Metabolites to Abiotic Stresses

Plants established several adaptive mechanisms to endure abiotic factors, containing variations of metabolism in various directions, to confirm their existence in combative environmental situations [66] (Table 2). Several plant metabolites could assist and reduce the effect of the harsh stress of salt, drought, and water by acting as osmolytes and osmoprotectants [67]. Examples of such metabolites include dimethylsulfoniopropionate (DMSP) and glycine betaine; sugars, such as sucrose, trehalose, and fructan; amino acids, such as proline and ectoine, as well as some metabolites of polyols, sorbitol, and mannitol [68,69]. In plants, a wide range of waxy layers known as epicuticular wax keeps water balance during water shortage and acts as a mechanical stoppage to encounter disease-causing agents. Additionally, ascorbic acids, glutamine, alpha-tocopherol, anthocyanins, and carotene shield plant tissues by foraging the intermediates of bustling oxygen produced during oxidative stress [70]. Similarly, several other smaller compounds guard plants against oxidation damage related to various constrictions [65].

Besides, the plant’s defense system is related to generating phytoalexins, stimulating the common phenylpropanoid pathway and producing lignin biosynthesis [71]. Further, phytochemicals and hormones such as salicylic acid and methyl salicylate, methyl jasmonate and jasmonic acid, as well as other small molecules formed due to stress, play a significant role against environmental stresses [72,73,74]. All of these may also function as signaling compounds by stimulating the resistance system and reactions of acclimation [75]. Among the defense systems of plants, osmotic regulation is one of the broadly pronounced responses to the water shortage that needs the accretion of harmonious solutes, such as sugars, amino acids, polyols, and glycine betaine [76]. These chemical compounds do a significant job in sustaining cell turgor and stabilizing cell membranes and protein. Moreover, other studies designate the importance of these compounds in rehabilitating redox stability through the scavenging of ROS, which could adversely affect cellular structures and metabolism [68,77].

**Table 1:** List of species, various metabolomics approaches, and applications cited in this review under diverse abiotic stresses.

Species	Abiotic Factors	Method	Application	References
*Arabidopsis thaliana* L.	Temperature	GC-MS	Exploring the temperatures stress metabolome	[58,78]
*Populus euphratica* Oliv.	Water and salinity	Metabolite profiling	Changes in early and late transcription and metabolite profiles	[79,80,81,82,83]
*Thellungiella halophila* (C.A.Mey.) O.E.Schulz		Metabolic fingerprinting	Identify metabolic changes in fruits	
*Solanum lycopersicum* L.		Metabolic fingerprinting	To classify control as well as salt-treated groups of tomatoes	
*Arabidopsis thaliana* L.		GC/MS and LC/MS	To reveal the short-term responses to salt stress	
*Arabidopsis thaliana* L.	Drought and flooding	Metabolic profiling	When defense pathways collide	[22]
			To identify the responses of plants to abiotic stresses	[84]
*Arabidopsis thaliana* L.	Sulfur	Multi-parallel, high-throughput analysis	To reveal novel findings	[85]
*Phaseolus vulgaris* L.	Phosphorus	Transcript profiling	To investigate global gene expression and metabolic responses	[86]
*Arabidopsis thaliana* L	Oxidative	GC-MS	To characterize the dynamics of metabolic	[87]
*Arabidopsis thaliana* L	Heavy metals Caesium (Cs) Cadmium (Cd)	NMR	Change metabolic consequences of stress	[88]
*Silene cucubalus* Wibel			Metabolomics analysis of the consequences of cadmium exposure	[89]
*Glycine max* L.	Salinity	GC-MS	Metabolomics analysis in the roots of different soya been varieties, under salinity levels	[90]
*Glycine. max* L.	CE-MS	CE-MS	Proteomic profile investigation of different soya bean varieties, under Cd stressed conditions	[91]

#### 4.1.1. Amino Acids

Amino acids are considered a precursor for protein and other organic molecules, e.g., nucleic acids, which designate an active part in the responses of a plant under several stress factors. Amino acids could also play a significant role in signaling and controlling molecules [92]. Various studies showed that many amino acids stored in plants are apparent to different abiotic stresses [93,94]. Moreover, the exposure of plants to such stresses appearance an accumulation of proline and other amino acids. In plants, the role played by stored amino acids differs after acting as an osmolyte to adjust ions passage, reducing stomatal opening and reclamation of TM [95]. Moreover, amino acids can also disturb the synthesis and activity of several enzymes, gene expression, and redox state of homeostasis [96]. The accumulation of proline and ectoine is considered the most extensively dispersed osmolytes, as they act as osmoprotectants to protect plants from harmful effects and exciting environmental stresses, including low and high temperature, salinity, UVr, water, and osmotic stresses [68,97]. 

Primarily, proline is produced from a glutamate and proline metabolizing enzyme, pyrroline-5-carboxylate synthetase (P5CS), which reduces glutamate to pyrroline-5-carboxylate (P5C). At last, from the reduction of P5C, this stress-responsive amino acid forms by pyrroline-5-carboxylate reductases (P5CR) [98]. In transgenic plants, the significant role of proline was established during osmotic stress. For example, overexpression of the *P5CS* gene in soybean increased proline content and, thus, tolerance to salt stress in transgenic plants [99]. Besides osmolytes, proline is thought to accomplish many other important functions related to plant resistance, e.g., ROS scavenging, redox balancing, cytosolic pH buffer, molecular chaperon, and a stabilizer of protein structure [98]. Subsequently, in response to abiotic factors, the enlarged levels of proline were observed for several years to be the stress-responsive feature in plants. The relationship between the accumulation of proline as osmolytes and stress tolerance had a great share because of its applicability to different crops [100,101]. 

Remarkably, some of the metabolites were related to drought resistance and drought vulnerability of the considered hybrids [102]. Additionally, studies on drought responses at metabolomics levels indicated that Andean potatoes with a phenotype designating greater stress exposure have more proline related to the genetically assembled plant that was a higher dearth-tolerant [103]. It was established that the cultivar with a sensitive phenotype has high-level certain amino acids, containing proline and Gamma-aminobutyric acid (GABA) when barley exposed to salinity stress [104]. It may well advocate a greater liability of these plants to such stress. According to [96], this accretion could be associated with the deterioration of the leaf and slowing the development of a more subtle genotype. Furthermore, studies on Arabidopsis revealed that proline could be a lethal compound under heat stress [105], while Charlton et al. found that water deficiency was the cause of the decrease in isoleucine concentration in *Pea* and *Arabidopsis* plants [106].

#### 4.1.2. Polyamines 

Plants are tested by different stress factors and adversely affect their growth, yield, and geographical circulation [107]. To survive the combative environmental stress circumstances, plants have developed many adaptive strategies, amongst which the accumulation of metabolites plays an important defensive role [108]. Metabolites strongly involved in stress resistance are the low-molecular-weight (LMW) acyclic polyamines [109]. Polyamines are the LMW nitrogen-containing organic compounds with more than two amino groups with a positive charge at the cellular pH, allowing them to link with negatively charged molecules, such as nucleic acids, phospholipids, and proteins [110]. Usually, polyamines are polycations essential for plant growth and development and play an important role in abiotic stress resistance in higher plants. Triamine spermidine, tetraamine spermine, and their diamine predecessor, putrescine, are the general polyamines [111]. Because of their cationic nature, these compounds have often been correlated to environmental stresses, such as drought, chilling, heat, TM, and salinity [112]. 

The results of Khan et al. [95] and Capell et al. [113] showed that the accumulation of spermidine with the up-regulation of spermidine synthase of *Cucurbita ficifolia* augmented several stress responses in a recombinant Arabidopsis plant, such as waterlogging and salinity stresses. It was shown that spermidine acts as a signaling molecule and controls the assertion of intricate genes in drought resistance. Furthermore, it has been demonstrated that polyamines are attributed to being involved in maintaining membranes shielding from damage under stressful environments [114] and controlling the formation of nucleic acid as well as enzyme activity [115]. Additionally, different findings revealed that polyamines play a significant role in oxidative stress by mitigating the balance state of ROS through their direct contact or indirectly regulating the antioxidant system and suppressing ROS production. Moreover, some authors hypothesized that polyamines could act as a cellular signal in plants throughout the stress responses [116]. 

#### 4.1.3. Carbohydrates

Carbohydrates produced during photosynthesis are the main building units that provide energy and support to the plant biomass [117]. Extensive studies revealed that non-living factors lead to the assemblage of non-structural saccharides, such as sucrose and lactose, simple sugars, or polyhydric compounds (alcohols and phenols), amongst various species of plants [118]. Particularly, there is a robust association between carbohydrate accretion and osmotic stress resistance, including oxidative stress (ROS) conditions, salt stress, and the scarcity of water [95]. As a source of carbon and energy in a cell, soluble carbohydrates may take a significant part in the metabolic processes of plants. Several stress factors may impact the level of these soluble carbohydrates because the accumulation of carbohydrates is associated with photosynthesis [119]. Rosa et al. [120] demonstrated that certain soluble sugars, such as sucrose and hexoses, improved stress tolerance by down-regulating the stress-related genes and up-regulating growth-related genes. Though, the contents of certain carbohydrates, such as raffinose, glucose, fructose, and maltose, are highly sensitive to environmental stresses and increase. However, the contents of myoinositol were reduced in barley roots during water-scarce conditions [121]. The findings of Sperdouli and Moustakas [122] revealed an increase and contact of augmented soluble carbohydrates, sustaining a great antioxidant defense in the leaves of *Arabidopsis thaliana* under dry environmental stress conditions. Studies showed renovation of carbon metabolism under salt-related stress (paraquat) in *A. thaliana* tissues and inferred by the researchers as a substitute approach to staying alive [122]. 

In water-deficit conditions, soluble sugars function as osmoprotectants, decreasing the harmful impact of osmotic stress and helps in sustaining the turgidity of cell and cell membrane stability by keeping plants from humiliation [123]. Under stress conditions, the increase in sugar quantity is generally the result of carbohydrate hydrolysis that needs enzymes with hydrolytic usage [124]. Moreover, carbohydrates that are soluble, such as disaccharides (sucrose and trehalose), oligosaccharides (raffinose and stachyose), and polymer of fructose molecules (fructans) next to their linked metabolic enzymes are essential compatible osmolytes associated with the scavenging of unstable molecules (ROS) during their assortment in plant tissues [125]. In low-temperature stress, sugar alcohols, such as polyols, function as osmoprotectants and shield cell membranes against ice adhesion [77]. Moreover, carbohydrates may act as signaling molecules [126]. The demonstrated data advocate a specific response of carbohydrates in plants. However, it should be noted that the accumulation of carbohydrates depends on the kind of stress to which it bared [127]. 

#### 4.1.4. Glycine Betaine

Glycine betaine (GB) is a widely studied quat compound, which is active in retaining the water balance between the plant cell and the environment during drought conditions. Moreover, GB playing a significant role in stabilizing the macromolecules, shielding photosynthesis, detoxification of reactive oxygen radicals, and as an osmoprotectant [128,129]. Several studies indicated their importance in improving plant tolerance under various abiotic factors. It has been shown that plants are distinguished according to the formation of GB, such as barley, spinach, maize, and wheat, produce and accumulate a higher quantity of GB in their chloroplast. However, some plant species cannot obtain substantial amounts of GB during stress, such as *A. thaliana*, rice, and tobacco [130]. Furthermore, it has been shown that transgenic plants could mitigate the impact of abiotic stresses. Therefore, efforts have been made to improve tolerance through glycine betaine biosynthesis to achieve transgenic plants. In transgenic plants, such as *Arabidopsis*, the *cyanobacteria* genes, such as glycine sarcosine methyltransferase, and in transgenic maize, a greater amount of GB accumulates. As a result, in transgenic *Arabidopsis*, resistance to drought and salt is greater; nevertheless, a recombinant plant of maize retained well in cold-related to non-transgenic cultivars [131,132]. 

Moreover, through genetic engineering, other transgenic plants with a GB-producing capacity have been achieved, including *Brassica juncea* and tobacco with greater tolerance to salt and chilling, indicating a progressive ability to propagate and grow well related to wild-type in abiotic environmental conditions [133,134]. Besides, transgenic tomatoes with GB synthesis were more resistant to cold stress and produced fruit at a rate from 10 to 30% higher than the wild type. [135]. Though, the meditations of GB produced in every transgenic plant were scarce to control the osmotic stress to which plants were exposed. Similarly, previous studies showed that GB could enhance root growth and reduce oxidative stress. Additionally, the exogenous application of GB improves the stress tolerance of Cr in chickpea plants [136] and salinity stress in wheat [137]. Consequently, further protecting approaches of GB, such as defense against ROS and heavy metals stress, should be considered, which may enhance the tolerance level [138].

#### 4.1.5. Lipids

Lipids are a fundamental component of biological membranes, particularly the plasma membrane, which serves as the contact between the cell and its surroundings [72]. Lipids can be grouped into eight major types based on the chemical structure in conjunction with distinctive hydrophobic and hydrophilic components, such as fatty acids, glycerides, phosphoglycerides, sphingolipids, steroids, isoprenoids, glycolipids, and polyketides [139]. Being sessile organisms, plants are subjected to a wide variety of biotic and abiotic factors, such as temperature, drought, heavy metals, salinity, and pathogen attack. However, lipid-mediated signaling occurs in response to all these stressors (Figure 2). The plasma membrane, which is typically the signaling source of lipids, is commonly used by plants to sense these stimuli and transform the signal into subsequent biochemical metabolism. Generally, these are acclimating enzymes that have all been proposed as signaling lipids, such as phospholipases, lipid kinases, and phosphatases [140]. Commonly, lysophospholipid, fatty acid, phosphatides, triacylglycerol, inositol phosphate, oxylipins, sphingolipids, and nacylethanolamine are considered the major contributing signaling lipids molecules [141]. The conformation and activity of cellular proteins and metabolites are influenced by signaling lipids because they have the ability to temporarily attract molecular markers to the membrane. 

The enzyme phospholipase A (PLA) is very important in the formation of fatty acids and lysophospholipids. Usually, lysophospholipids are present in very limited amounts in plant tissues; however, in stressed conditions such as freezing their quantity increases [142]. Some reports revealed the physiological role of lysophospholipids against various environmental stresses. Similarly, the phospholipase A2 (PlA2) has been shown to increase the production of some elicitors in poppy plants [143], while lysophosphatidyl-choline and lysophosphatidyl-ethanolamine act as signals transducers in arbuscular symbiosis in potato [144].

Fatty acids have also been demonstrated as stress-responsive lipids in plants. Oleic acids modulate nitric oxide-related proteins, thereby regulating nitric oxide and mitigating tolerance in *Arabidopsis* [145]. Moreover, fatty acids also regulate drought, salt, and heavy metals tolerance, as well as the wound-induced responses of pathogens/herbivores in plants [146]. Likewise, the responsive role of phosphatidic acid (PA), inositol polyphosphates, oxylipins, sphingolipids, and some other lipids have been studied in various plant species [147,148,149]. Some of the environmental stress factors under which the plant lipid responses were reported to include chilling, freezing, and wounding [150], pathogens [151], low-temperature stress [152], salt stress [153], and water and drought [154] stress response.

### 4.2. The Response of Secondary Metabolites to Abiotic Stresses

Primary metabolites are compounds that are related to important physiological functions in organisms. Hence, they are generally found in all plant species and are directly involved in growth, development, and reproduction [155]. Compared to primary metabolites, secondary metabolites are very definite in their function, as they are not directly involved in plant growth, development, and reproduction of organisms. Generally, they are species-specific that could be redundant in different situations [156]. Usually, they are made under particular conditions for a definite purpose, such as defense against pathogens infection, enhanced resistance to abiotic stresses, and protect the harmful effect of UVr [157]. Furthermore, secondary metabolites produce different compounds important for several biochemical and biophysical processes in plant cells and tissues (Table 2). However, they have no common familiar physiological functions in plants, such as photosynthesis, respiration, translocation, transportation of solute, acclimatization of nutrients, and differentiation [158].

In addition, the specified plant species produce these natural products, and their concentration level is controlled to some extent with the growing period, environments, and adjustment progress [159]. Substantially, they attracted insects and animals for fertilization and seed spreading. The accumulation of phenyl amides in beans to the impact of abiotic factor (heat) was described, proposing an antioxidant role of these secondary metabolites [160]. Modern research tries to identify the key roles the secondary metabolites play in plants as indicators, antioxidants, and for other purposes. Secondary metabolites are also important in plants used by humans [161]. Besides, the compounds of secondary plant metabolites are distinctive means of food essences, medicines, flavorings, and other industrial materials [162]. In plants, the accretion of certain metabolites frequently occurs exposed to different stress factors, such as several phytohormones, elicitors, TM, and signal transduction compounds [163,164,165]. 

**Figure 3:**
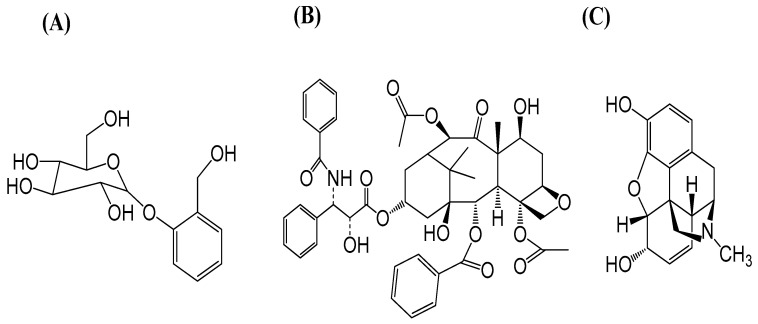
Some eminent examples with medicinal properties of secondary plant metabolites are (**A**) salicin, (**B**) taxol (paclitaxel), and (**C**) morphine.

Some famous examples of secondary plant metabolites with medicinal properties include the anesthetic and antipyretic compounds salicin taken from *Salix* sp., which is used to make aspirin [166]. Similarly, other pharmacological secondary metabolites, such as taxol (anticancer), sequestered from pacific yew (*Taxus brevifolia*), and the strong obsessive compound morphine removed from opium (*Papaver somniferum*). Secondary metabolites have the following three major groups: phenolics, terpenes, and S and N comprising compounds (Figure 3) [167,168]. 

#### 4.2.1. Phenolic Compounds

In plants, phenolic compounds are recognized as the largest and essential group of secondary metabolites changing from simpler aromatic rings to more complicated ones, such as lignin, and play a significant physiological role in increasing the resistance and adaptableness suboptimal circumstances during the life cycle of plants [169,170]. Phenolics are produced in optimum and sub-optimum environments in plants and play a major role in various developmental mechanisms, such as cell division, balancing hormones, photosynthetic processes, and reproduction, as well as in the mineralization of nutrients [75]. These compounds constitute secondary metabolites, including lignins and tannins, flavonoids, isoflavonoids, anthocyanins, and coumarins [171]. Moreover, all these chemical compounds are produced in plants by the phenylpropanoid pathway, in that phenylalanine compound is the main substratum that can do significant work in the resistance mechanism of plants against various stress factors in the environment [172]. 

The pathway of phenylpropanoid is regulated by biotic and abiotic factors, including drought, salt stress, TM, low or high temperature, wounding, pathogen attack, herbicide treatment, nutrient deficiencies, and UV radiations causing the accumulation of different phenolic compounds [75,173]. Consequently, the aggregations of phenolic compounds in plant materials are considered an important sustaining strategy of plants in harsh environmental situations. Hence, respond to these stresses and contribute to the removal of ROS, catalyzed-oxygenated reaction with the establishment of metabolic structures and obstructing the processes of oxidative enzymes, thus increasing evolutionary aptness [174,175]. Besides, phenolic accumulation is also considered a reliable feature and key defense mechanism under stress, leading to the enhanced creation of free radicals and other oxidative species in plants [176]. 

Moreover, to survive in oxidative stress conditions, plants had established two diverse biological ways, such as escaping ROS creation and eliminating it through enzymatic and non-enzymatic processes, such as the deposition of LMW antioxidants [177,178]. Further, studies revealed that the accumulation of LMW antioxidants results due to the activities of phenylalanine ammonia-lyase (PAL), chalcone synthase (CHS), and other essential enzymes [179]. Various physiological processes of plants related to growth and development in plants comprising seed germination, cell division, and synthesis of photosynthetic pigments, are influenced by phenolic acids and flavonoid accumulation for persistence and adaptation to environmental conflicts [180,181]. In particular, phenolic compounds consult greater tolerance in plants such as TM stress, which enhances the production of ROS and reduced growth [182], and phenolic compounds (flavonoids), in response, protect plants from oxidative stress damage through the chelation process [183,184]. Similarly, when plants are exposed to other abiotic factors can also affect their life cycle. Under drought conditions, the concentration of ferulic acid decreased, while the p-coumaric acid and caffeic acid increased in maize xylem sap, which could be supportive in stiffening and lignification of the cell wall [185]. Spatially confined fluctuations in cell wall phenolics were presented to be engaged in the advanced inhibition of wall extensibility and root growth, which can enable root acclimation to drought [186]. 

Various environmental stress factors mediated the synthesis of flavonoids, isoflavonoids, and anthocyanins. In plants, flavonoids play a defensive role due to their antioxidant properties when exposed to a water-deficit situation [187]. Moreover, Nakabayashi et al. [188] indicated that flavonoid significantly improves resistance in *A. thaliana* in water scare conditions. Similarly, phenolic acids and flavonoids as antioxidants and sunshades are involved in plants’ response to a dry environment [189]. Other studies reported that different polyphenolic was associated with gene expression reforms in an account of potato plants under drought conditions, though the fluctuations were greatly specific to the cultivar [190]. Rodziewicz et al. [96] and Parida et al. [191] suggested that polyphenols are involved in conserving osmotic potential in cells and confiscating free radicals during drought stress. Besides, polyphenols affect the source and movement of organic and inorganic soil nutrients existing for plants and microbes and indicate a reply to nutrient insufficiency, therefore offering a way for identifying nutrient ailments earlier to the occurrence of evident symptoms [192]. Stress conditions of drought and waterlogging increased the flavonoids quercetin and rutin in the herbaceous pharmaceutical plant *Hypericum brasiliense*, whereas cold stress caused a different reaction [193]. Comparatively, a greater decrease in flavonoids was noticed in the sensitive genotypes; thus, they could show that the flavonoid content was imperative in sustaining the greater antioxidant activity in water-stressed conditions [96,194]. 

Furthermore, anthocyanins were identified to increase their content in plant tissues against drought and cold stress because of their antioxidant and ROS scavenging properties, which cause protection to plant cells [195,196]. In red-fleshed apple callus culture, low temperature (16 °C) tempted an increased level of anthocyanin [197]. 

Additionally, the assembly of phenolics rises into the cell wall either as suberin or lignin under low-temperature stress [198]. Though, suberin deposition and lignification increase the adaptability and resistance to cold stress [192]. Similarly, to respond to the negative effects of Uvr, endogenous phenolic compounds (flavonoids) accumulate in plant cells and make a shield under the epidermal layer, which protects the plant and the component of the cell from these harmful radiations [199]. Moreover, flavonolignan silymarin has been reported to accumulate in in-vitro cultures of *Silybum marianum* upon application of abiotic stress treatments, such as NaCl, polyethylene glycol, and gamma irradiation, because of the defensive mechanisms that cells perform to counteract the stress of these factors [200]. Therefore, stress elicitation successfully produces high-value phytoconstituents from medicinal crops [201,202].

#### 4.2.2. Terpenoids

Plants have developed a complex resistance system that depends on the swift perception and instigation of secondary metabolites to adopt different environmental stress factors in an ecosystem [203]. Among all, terpenoids establish a broad and structurally diverse group of lipophilic secondary metabolites, which are produced in plants from isoprene units (C_5_H_8_) [204]. Physiologically, terpenoids play an important role as phytohormones, such as the sesquiterpenoid abscisic acid (ABA) and the diterpenoids gibberellic acid (GA), against biotic and abiotic stresses. Studies have shown that the phytohormone abscisic acid (ABA) triggers defense mechanisms, such as facilitating responses to drought and water stress by adapting the membrane properties [205]. Moreover, terpenoids show antioxidant and antibiotic activity that maintains lipid membranes and increases environmental stress tolerance against herbivores [206]. 

Terpenoids also function as phytoalexins (LMW antimicrobial compounds), prepared as part of the plant defense mechanism in response to abiotic and biotic factors. For example, many diterpene phytoalexins have been reported in *Oryza sativa* [207]. Similarly, in cotton plants, sesquiterpenoid phytoalexins, such as gossypol, hemigossypolone, and heliocides, as defensive metabolites accumulated both above and below the ground against pathogens and herbivores [208]. Moreover, in maize and rice leaves and roots, diterpene phytoalexins are produced, including zealexins, kauralexins, and oryzalexins that exhibit antimicrobial properties and respond against pathogenic fungal blast diseases, such as rice blast caused by *Magnaporthe grisea* [207,209]. Additionally, UVr and TM stress induced the accumulation of rice phytoalexins. According to Vaughan et al. [209], the accumulation of phytoalexins in response to drought is root-specific and does not affect the level of phytoalexins aboveground. However, the reduced content of the terpenoid compound was described in cotton species in drought conditions [191,210]. Yusuf et al. [211] noticed that the increased content of soluble alcohol tocopherol with antioxidant properties shows a significant role in the mitigation of stress by stabilizing the cell membranes induced by salinity, TM, and osmotic potential in *B. juncea*. Furthermore, the content of saponins in soybean plants was recognized as one of the crucial secondary metabolites related to the resistance of salt stress [212]. 

**Table 2:** The response of various types of metabolites against different abiotic stresses.

Metabolomics	Stress	Mode of Action	References
Primary Metabolites			
Amino acids: (proline)	Drought, salinity, temperature, and cold	Acts as osmoprotectant	[77,96,97]
Polyamines: (triamine spermidine, tetraamine, spermine)	Heavy metals	Regulating antioxidant systems, suppressing ROS production	[168]
Carbohydrates: a. (sugar, sucrose)	Water deficit	Osmoprotectant, maintain turgor, cell membranes stability	[95]
b. alcohols (sorbitol, ribitol, and inositol)	Cold stress	Cryoprotectants protect cell membranes against ice adhesion	[97,124]
c. disaccharides, raffinose	ROS	ROS scavengers, control ROS signaling	[39,106]
Glycine betaine	Drought, ROS, salt, and low temperature	Osmoprotectant detoxification of ROS,	[128]
Lipids	Heavy metals stress	Scavenge the ROS production	[136,138]
Secondary Metabolites			
Phenolic compounds: p-coumaric acid, caffeic acid; flavonoids, anthocyanin, suberin, or lignin	Heavy metals/ ROS	Scavenging of ROS and chelation process	[151,159]
	Water stress	Antioxidant mechanism	[154]
	Drought, UV	Stiffening and lignification of the cell wall, antioxidant, and sun shields properties	[191]
	Drought, nutrient deficiency	Scavenging of ROS, maintenance of osmotic potential in cells, and identifying nutrient ailments	[77]
	Cold and drought	Increase resistance and protect plant cell	[161]
	Cold or low temperature	Lignification and submarine deposition increase adaptability and resistance	[213]
TerpenoidsAbscisic acid (ABA), gibberellic acid (GA), phytoalexins (gossypol, hemigossypolone and heliocides), momilactones, oryzalexins, tocopherol, saponins	Biotic and abiotic factors	Physiological function, ameliorate heavy metal stresses, antioxidant, and antibiotic activity	[170,171]
	Heavy metal, drought, UV, pathogens, and herbivores	Improve stress tolerance, drought, heavy metals, and enhances antimicrobial properties	[207,208,209,210]
	Fungal blast	Stabilizing the cell membranes	[212]
	Salinity, heavy metal, potential osmotic	Salt stress tolerance	[212]
Nitrogen-containing metabolites Alkaloids Glucosinolates Non-protein amino acids	Drought, herbivores	Increase tolerance level and defense against herbivore attack	[158,214]
	Drought, waterlogging	Osmoprotectants increased phytochemical contents	[215,216]

#### 4.2.3. Nitrogen-Containing Secondary Metabolites

**Figure 4:**
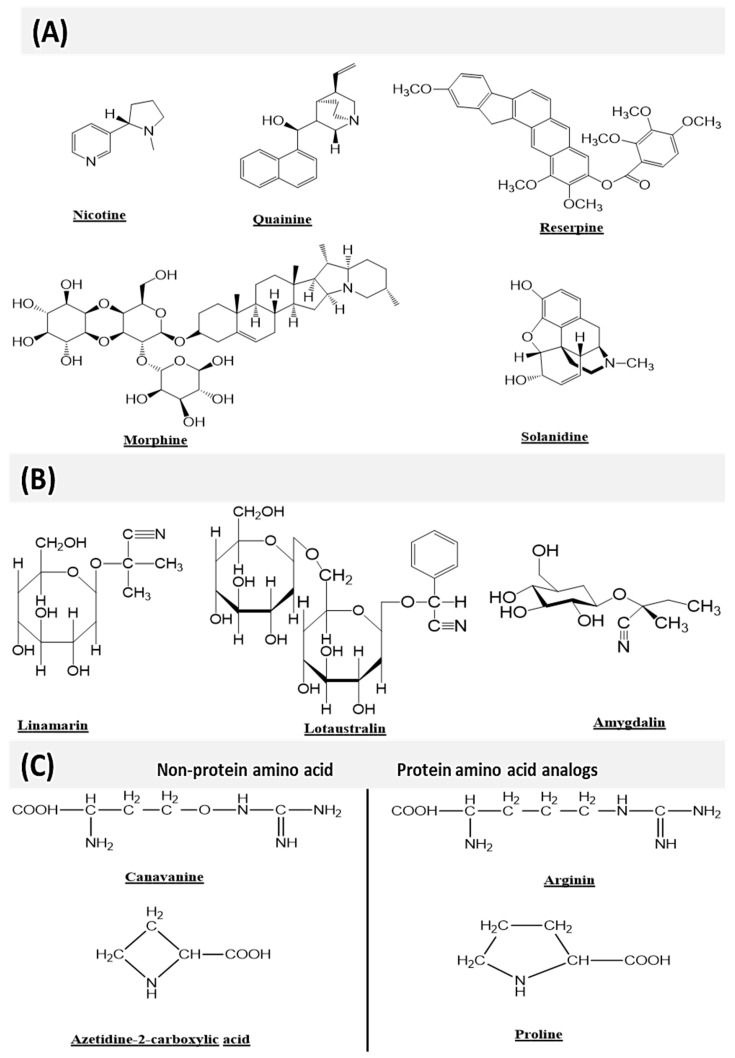
Chemical structures of some plants derived primery and scndary metabolites with key importance in different era of lfe. Among all, some commonly known alkaloids (**A**), cyanogenic glycosides (**B**), and (**C**) non-protein amino acids along with their protein amino acids analogues.

Plants have developed several defense mechanisms against invading enemies, such as microbial pathogens and herbivorous animals, as well as abiotic factors, e.g., drought, waterlogging, and salinity, which are considered for the high loss of crop production worldwide [217]. However, plants have developed a complex defense system of secondary metabolism against these stressors, including the nitrogen-containing secondary metabolites, such as alkaloids, cyanogenic glycosides or glucosinolates, and non-protein amino acids (Figure 4) [158,217]. Previously, nitrogen-containing secondary metabolites were considered unwanted materials of plants and are known now for their resistivity towards different stress factors [119]. Among the phytochemicals, alkaloids are heterogeneous groups of secondary metabolites consisting of one or more nitrogen atoms produced under abiotic stress conditions. It has been found that alkaloids perform a significant role against microbial pathogens and herbivorous animals. Besides, more alkaloid contents and derivatives are produced in abiotic stress conditions. For example, poppy plants make more alkaloids when there is a drought period as well as under salinity stress [214]. In lupins (*Lupinus termis*) cultivars, the content of alkaloids was also influenced by the drought and activated yeast extract treatment [216]. 

Additionally, glucosinolates and cyanogenic glycosides are sulfur and nitrogen-containing secondary metabolites derived from glucose and amino acids. Similarly, Rodziewicz et al. [96] demonstrated that all these natural compounds play a significant role against different environmental factors (biotic and abiotic). Mewis et al. [215] showed that in *A. thaliana*, under drought and water logging conditions tend to increase aliphatic compounds of glucosinolate and flavonoids. Moreover, in *B. juncea,* the increased level of glucosinolate was observed during the vegetative stage under water deficit conditions. In plants, apart from the essential 20 amino acids, there are more than 200 free plant cell amino acids that are not assimilated into proteins. These free amino acids are called non-protein amino acids. Their major function in plants is to respond to various environmental stresses [158,217].

## 5. Conclusion and Future Perspectives

The acceleration of climate change increases the severity of damage to crop productivity under environmental stress. Understanding the role that primary and secondary metabolites play during stress resistance mechanisms is important for developing crop species and improving their stress resistance, ensuring that the need for food security is met for a growing global population. However, less has been understood about the function of these metabolites against environmental stresses in plants, especially abiotic stresses. In the current review, we have provided an overview of the role of primary (amino acids, polyamines, carbohydrates, glycine betaine, and lipids) and secondary (phenolics, flavonoids, terpenoids, alkaloids, and glycosides) metabolites against several abiotic factors, such as drought, salinity, temperature, UVr, and TM. Analysis of more than 200 articles allowed us to describe the main responses of primary and secondary metabolite products of different plant species to abiotic stresses. Metabolomics has occupied a prominent place in plant stress physiology and biology research. Metabolic change due to abiotic stress is complex to describe the variability between different plant species. Nevertheless, metabolomics needs more extensive research in data annotation, assessment, processing, and evaluation. Progress in “omics” tools and bioinformatics and enhanced assimilation of the data from varying molecular levels is needed. Hence, to expose the full picture of sustaining mechanism, which will lead to new biomarkers of resistance towards biotic and abiotic stresses. Affirmation of the impact of environmental stresses on plants and their metabolite level responses recorded valued genes about the mechanism underlying such acclimation. However, the balancing mechanism between the gene expression and the subsequent metabolic phenotype is a big challenge nowadays. Therefore, comprehensive research of the dynamic behavior of metabolic systems is a great task for researchers in systematic biology. Furthermore, identifying the genetic background behind the diversity of primary and secondary metabolites produced by plants will help in improving and developing stress tolerance. Manipulating and overexpressing genes related to the biosynthetic pathway of secondary metabolites could be a solution for plant tolerance to environmental stress conditions.
